# Aromas Influencing the GABAergic System

**DOI:** 10.3390/molecules27082414

**Published:** 2022-04-08

**Authors:** Neville Hartley, Craig S. McLachlan

**Affiliations:** 1 Department of Naturopathy and Western Herbal Medicine, Health Faculty, Fortitude Valley Campus, Torrens University Australia, Brisbane, QLD 4006, Australia; 2 Centre for Healthy Futures, Health Faculty, Surry Hills Campus, Torrens University Australia, Sydney, NSW 2010, Australia; craig.mclachlan@torrens.edu.au

**Keywords:** aromas, essential oils, volatile chemicals, constituents, GABAergic, GABA_A_ receptor, anxiolytic, sedative

## Abstract

Aromas have a powerful influence in our everyday life and are known to exhibit an array of pharmacological properties, including anxiolytic, anti-stress, relaxing, and sedative effects. Numerous animal and human studies support the use of aromas and their constituents to reduce anxiety-related symptoms and/or behaviours. Although the exact mechanism of how these aromas exert their anxiolytic effects is not fully understood, the GABAergic system is thought to be primarily involved. The fragrance emitted from a number of plant essential oils has shown promise in recent studies in modulating GABAergic neurotransmission, with GABA_A_ receptors being the primary therapeutic target. This review will explore the anxiolytic and sedative properties of aromas found in common beverages, such as coffee, tea, and whisky as well aromas found in food, spices, volatile organic compounds, and popular botanicals and their constituents. In doing so, this review will focus on these aromas and their influence on the GABAergic system and provide greater insight into viable anxiety treatment options.

## 1. Introduction

The sense of smell with aromas is common in everyday life, and these aromas can elicit neurological, cognitive, or behavioural responses [1]. For example, it is not uncommon for a smell to evoke a previous visual memory [2]. Aromatic plants and oils have been used as incense, perfumes and cosmetics, for medicinal and culinary purposes, and in religious rituals since time immemorial. Sprigs of juniper are still burnt in Tibetan temples in the East for purification, as are frankincense used during Roman Catholic mass in the West [3] (p. 11).

In recent times, studies have revealed inhalation of certain aromas exerts psychophysiological effects on humans [4]. Numerous animal and human studies have demonstrated anti-anxiety effects from the inhalation of various aromas [5,6,7] and other studies have elucidated the purported mechanism of action of these aromas exerting their anxiolytic effects [1]. 

Aromas are volatile chemicals < 300 Da that are detected by the olfactory system [4]. Aromas first dissolve into the mucus lining of the nasal cavity and then bind to olfactory receptors in the olfactory epithelium, generating an action potential within the receptor neuron [2,4]. An electrical signal is created and sent to the olfactory bulb for primary processing and then for final processing in various brain regions such as the amygdala, hippocampus, orbitofrontal cortex, and thalamus [2]. The olfactory neuroanatomy is intertwined with the primary emotional areas, including the amygdala, hippocampus, and orbitofrontal cortex via extensive reciprocal axonal connections [7]. It is also thought that these aromas may exert direct effects on neuronal receptors in the brain by crossing the blood-brain barrier [4].

The brain regions most crucial in regulating negative emotions such as anxiety are a set of limbic structures with the amygdala being a focal point [8]. A common finding from a variety of clinical anxiety disorders including social anxiety disorder, post-traumatic stress disorder (PTSD), obsessive compulsive disorder, phobias, and panic disorder is hyperactivity of the amygdala in response to negatively valanced stimuli [9]. Of particular interest is the brain GABAergic system, which is central to the regulation of anxiety. γ-aminobutyric acid (GABA) is a major inhibitory neurotransmitter in the central nervous system (CNS) and is said to be utilised by one-third of CNS neurons as their primary neurotransmitter [8]. GABAergic neurotransmission in the amygdala is important in regulating anxiety-related behaviours [8]. For instance, administration of benzodiazepines reduces amygdala activation in the presence of negative emotional stimuli. In addition, infusions of GABA or GABA receptor agonists into the amygdala decreases measures of anxiety in several animal species [8]. The hippocampus is another limbic structure that has reciprocal connections with the amygdala, projects to the hypothalamus affecting the release of adrenocorticotropic hormones, and has been implicated in dementia [10], anxiety disorders, and PTSD [11]. Animal studies using dementia models have revealed early loss of GABAergic interneurons resulting in hippocampal hyper-excitability, and neuroimaging studies in patients with dementia have reported hyper activity in the hippocampus [12]. Many dementia patients experience high levels of anxiety. In addition, several molecular and biochemical changes in the GABAergic system have been reported in the dementia brain, in particular a reduced expression of GABA_A_ receptors in hippocampal neurons [13]. Thus, the GABAergic system undergoes significant remodelling in the dementia brain, and administration of exogenous agents that bind to GABA receptors may prove a useful tool in inhibiting typical GABA-related phenotypes, such as anxiety [14]. 

Neuronal inhibition of GABA is mediated via GABA receptors with GABA_A_ receptors being important in controlling excitability of the brain and modulating anxiety [15]. GABA_A_ receptors are a superfamily of pentameric ligand-gated ion channels that are widely distributed in the brain [15], consisting of five subunits arranged pseudo-symmetrically around a central pore with each subunit comprising of an extracellular, intracellular, and transmembrane domain [16]. The five subunits are made up of two α, two β, and one γ subunit, typically γ2, as approximately 75–80% of all GABA_A_ receptors contain γ2 [17]. Each one of the subunits has a distinct cellular and reginal distribution in the brain with some cell types expressing few, most, or all GABA_A_ receptor subunits [15]. Two binding pockets for GABA in GABA_A_ receptors are formed at the extracellular interface between adjacent α and β subunits. The pockets are formed by loops A-C of the β subunit at the principle side and loops D-F of the α subunit at the complementary side [15]. Benzodiazepines are a class of anxiolytic compounds that bind to GABA_A_ receptors at the interface between adjacent α and γ2 subunits enhancing flux of GABA-induced chloride ions, resulting in neuronal hyperpolarization and allosteric modulation of these receptors [15,17]. GABA_A_ receptors are chloride ion channels that open in response to GABA and are influenced via medications such as benzodiazepines, barbiturates, and, more recently, via inhaling certain aromatic compounds. 

### 1.1. Anxiety Assessment: Animal Behavioural Models

Animal studies have used various behavioural modeling to assess anxiety and the involvement of the GABAergic system. The elevated plus maze is a widely used behavioural model that has predictive validity. It assesses anxiety responses in rodents and relies on the proclivity of rodents toward dark, enclosed spaces and an avoidance to heights and open spaces [18,19]. Anti-anxiety behaviour is demonstrated by increased open arm exploration–percentage of time spent and entries [19]. The elevated plus maze is considered an etiologically valid animal model of anxiety because it uses natural stimuli [20] and is generally accepted to be specifically suited to evaluate anxiolytic substances that act via the GABA_A_-benzodiazepine receptor complex [21,22]. The light-dark box, open field, hole-board, marble-burying, and social interaction tests are other well-known tests to assess anxiety. The light-dark box test is commonly used for assessing unconditional anxiety-like behaviours in rodents and is based upon the conflict to explore novel areas and the aversion to open and brightly lit spaces [23]. Increased number of crossings between the light and dark compartments as well as increased time spent in the light compartment reflects anti-anxiety behaviour [23]. The open field test systematically assesses general locomotor activity and novel environment exploration and provides an initial screening for anxiety-related behaviour [24]. Anxiety-like behaviour in the open field is influenced by socially isolating animals from their cage mates when performing the test and the stress caused by the brightly lit, unprotected, and novel environment [24]. Typically, anxious rodents will spend more time on the periphery or along the walls (thigmotaxis) of the enclosure compared to time in the centre. Common outcome measures to gauge anxiety include activity time within the first five minutes, centre time, and defecation [25]. Moreover, time in the centre is generally sensitive to acute GABA-acting anxiolytics such as benzodiazepines [25]. 

The hole-board test originated from the open field test and remains one of the standard procedures applied in psychopharmacology and behavioural studies [26]. It offers a simple method to evaluate an animal’s response to an unfamiliar environment and has been used to assess anxiety and or responses to stress [27]. Head-dipping in the holes is considered the key feature of this behaviour assessment [26] with increased or decreased head-dipping indicative of anxiolytic or anxiogenic effects, respectively [27]. The marble-burying test is commonly employed as a model or measure to study anxiety-like and compulsive-like behaviour or anxiolytic or anticompulsive drug action [28]. Under anxiety or conditions of stress, rodents bury the marble as a natural defence mechanism, and marble burying is used as an index for anxiety disorders [29]. The social interaction test uses ethologically relevant sources of anxiety and uses the natural form of behaviour as the dependent measure [30]. Compounds acting on the GABA and serotonin (5-HT) systems have been extensively investigated across a number of behavioural models, which has played a vital role in unravelling the neural basis of anxiety. An increase in social interaction, without an accompanying increase in motor activity, is indicative of anxiolytic effects, whereas a decrease in social interaction indicates an anxiogenic effect [30]. 

### 1.2. Anxiety Assessment: Human Testing 

In human studies, various anxiety models of assessment have been employed, including the Spielberger State-Trait Anxiety Inventory (STAI), Anxiety Visual Analogue Scale (Anxiety-VAS), Visual Analogue Mood scale (VAMS), Profile of Mood States (POMS), and Humor Analogue Scale (HAS). The STAI is one of the most long-standing and commonly used self-rating scale for measuring anxiety severity. It measures the intensity of how much anxiety a person feels “right now, at this moment” (STAI-State) and the frequency a person “generally feels” anxious (STAI-Trait) [31]. Two 20-item scales are used to determine the level of state or trait anxiety with each item scored from one to four points (not at all, somewhat, moderate, and very much), with higher scores indicating greater anxiety [32]. The anxiety-VAS is a self-evaluation scale that is simple and rapid, consisting of a horizontal line 10cm long labelled “no anxiety” to the left and “worst possible anxiety” to the right [33] scored, by measuring the distance from one end of the scale to the subject’s mark on the line [34]. The VAMS is another useful tool to measure mood that is simple, promotes high compliance, and is both valid and reliable [35]. The VAMS is a unique assessment method that provides an assessment of eight clinically relevant, specific, internal mood states—sad, angry, afraid, confused, happy, tense, energetic, or tired—and is used to monitor the progress of an intervention on positive or negative mood states [36]. The POMS is a multidimensional self-report psychological instrument assessing short-term mood states, which are understood to be frequently fluctuating and transient [37]. The POMS 65-item is the most commonly used and represents six subscales assessing tension-anxiety, depression, anger-hostility, vigor-activity, fatigue, and confusion-bewilderment. Respondents rate each item on a scale from “0” (not at all) to “4” (extremely) reflective of the respondents mood over a period of time [37]. Lastly, the HAS is a self-assessment scale to accurately measure levels of anxiety, discomfort, sedation, and cognitive impairment [38]. It uses a 16-item rating battery with a 10 cm line and the extremes of feeling placed on either ends, where subjects mark an ungraduated 10 cm line to express the current emotional state of their respective feelings on each dimension [39]. 

The Frankl’s Behaviour Rating Scale (FBRS) and Facial Image Scale (FIS) are used to assess pediatric behaviour and anxiety. FBRS is a widely used behavioural evaluation scale in pediatric dentistry and research that evaluates a child’s attitude during dental treatment. It consists of four behaviour categories ranging from definitively negative to definitively positive [40]. FIS is a valid means of assessing dental anxiety in children and comprises a row of five faces ranging from very happy to very sad. Children point at a face they most felt like at that moment and a value is given to each face on a scale from one to five [41]. 

### 1.3. Neurochemical and Pharmacological Testing 

Various pharmacological substances have been used to explore GABA interactions, including flumazenil, 3-mercapto-propionic acid (3-MPA), GABA transaminase, and pentobarbital. Flumazenil is a benzodiazepine analogue that binds to the extracellular surface of GABA_A_ receptors and competitively displaces benzodiazepine, preventing further benzodiazepine binding [42]. Essential oils may also have GABAergic activity. To determine whether the anxiolytic-like activity of an essential oil occurs through the GABAergic system, flumazenil, a specific GABA_A_ receptor antagonist, is co-administered [43]. The ability of flumazenil to reverse the anxiolytic-like activity of an essential oil strongly suggests a role of this receptor complex in mediating its activity [43].

3-mercapto-propionic acid (3-MPA) is a known inhibitor of glutamic acid decarboxylase (GAD), an enzyme that reduces available GABA concentration and directly alters inhibitory transmission mediated by GABA. GABA is derived from glutamate via the action of GAD. The ability of a drug to block or inhibit 3-MPA-induced seizures is a well-established method for evaluating drugs and their effects on the GABAergic system [44]. GABA transaminase is a both a key synthetic and degradative enzyme that acts to maintain the transmitter pool of GABA [45]. Inhibition of GABA transaminase has been shown to increase brain GABA levels and properly preserve GABA concentrations [46]. 

The barbiturate pentobarbital binds to GABA_A_ receptors and, depending on its concentration, has the ability to modify activity in various ways, such as potentiating GABA_A_ channel activity at levels that are interacting significantly with the receptor but still at sub-full occupancy levels, e.g., less than ~100 mM [47]. 

This theoretical review summarises the effects inhaled aromas and their constituents have on the GABAergic system with reference to their anxiolytic and sedative properties. 

While not a systematic review, nonetheless an electronic search was performed using the online databases MEDLINE (PubMed), Scopus, Web of Science, Science Direct, and Google Scholar. The following key search terms or a combination of them were used: ‘aroma’, ‘aromatherapy’, ‘odour’, ‘inhalation’, ‘essential oils’, ‘GABA’, ‘gamma-aminobutyric acid’, ‘GABAergic’, ‘anxiolytic’, and ‘anxiety’. Additionally, individual essential oils with both common names and Latin binomial names were searched for. A final search was conducted using the reference lists of all relevant articles for additional studies to aid a complete theoretical review of the topic.

## 2. Aromas and Their Constituents

Aromas are volatile chemicals < 300 Da that are detected by the olfactory system [4]. Essential oils are a complex mixture of volatile and semi-volatile compounds stored in the glands, trichomes (glandular hairs), resin ducts, and oil ducts of plants. They are responsible for the specific aroma and flavor of plants and constitute up to 100 different metabolites [48]. A summary of the studies on inhaled aromas associated with GABAergic activity are shown in Table 1. The inhalation duration, the subject(s) (animal species or human demographic), intervention(s), observed effect(s), motor activity, and mechanism of action are included. Aromas and their constituents are reviewed below for evidence regarding their GABAergic effects. 

### 2.1. Acorus gramineus

*A. gramineus* is a perennial plant found in China, Japan, India, and Korea. The stems and roots contain 0.5~0.8% essential oil, and its main components are GABA, asarone, calamenol, palmitic acid, phenol, and palmitin [109]. In Chinese and Korean pharmacopeias, extracts of *A. gramineus* have demonstrated sedative, analagesic, digestive, diuretic, and antifungal effects [110]. Koo et al. [46] evaluated the central nervous system (CNS) effects of *A. gramineus* essential oil via fragrance inhalation in mice. Preinhalation of *A. gramineus* essential oil inhibited brain GABA transaminase activity, a GABA-degrading enzyme. The effect was influenced by the length of inhalation exposure (2 g of fragrance oil). In addition, there was both an increase in brain GABA levels and pentobarbital-induced sleeping time, which was progressively prolonged following *A. gramineus* essential oil inhalation. The barbiturate pentobarbital binds to GABA_A_ receptors and, depending on its concentration, has the ability to potentiate GABA_A_ channel activity [47]. These findings demonstrate sedative actions from fragrance inhalation of *A. gramineus* essential oil, which is thought to act on the CNS via GABA_A_ receptors. Liu et al. [111] have also shown that α-asarone, one of the main components of *A. gramineus,* when administered orally has anxiolytic activity in the elevated plus maze, marble burying, and light-dark tests, and Huang et al. [112] have demonstrated α-asarone to have GABA_A_ receptor modulation activity. 

### 2.2. Alpinia zerumbet

*A. zerumbet* (Pers.) B.L. Burtt & R.M. Sm, also known as shell ginger or *yan-shan-jiang* in China, is native to East Asia and Brazil and widely cultivated in tropical and subtropical zones of the world [113]. The leaves have been used in tea, and some research supports an association with life extension in Japan [49]. The essential oil obtained from the leaves of *A. zerumbet* have also been used to treat stress, anxiety, and depression. Murakami et al. [50] investigated the anxiolytic-like effects of *A. zerumbet* essential oil aroma in mice. Mice treated with *A. zerumbet* essential oil (0.0087 ppm and 8.7 ppm) demonstrated anxiolytic-like effects in the elevated plus maze. Satou et al. [114] support these findings with positive anxiolytic effects observed in all behavioural assessments in mice exposed to *A. zerumbet* essential oil, with the most significant effects seen in the elevated plus maze. Tissue distribution (blood, liver, kidney, and brain) of the four main constituents of *A. zerumbet* essential oil (α-pinene, p-cymene, 1,8-cineole, and limonene) were also assessed. Surprisingly, 1,8-cineole, the main component of *A. zerumbet* essential oil, was not detected in any tissues after *A. zerumbet* essential oil inhalation. The rationale for this is thought to be due to the monoterpene oxide rapidly metabolising. α-pinene accumulated in the brain at a similar rate to the liver and all components accumulated in the kidneys in the largest concentration. None of the compounds were detected in the blood; however, it is thought that the components rapidly shifted to the liver, kidney, and brain or were bound to protein in the blood. Thus, the main constituents of *A. zerumbet* essential oil are not necessarily distributed to organs throughout the body in the same proportions following inhalation [114]. Interestingly, while monoterpenoids may be rapidly metabolized, they are interesting in that they are composed of acyclic (open-chain), monocyclic, and bicyclic structures. There is evidence that monterpenoids can activate GABA_A_ receptors in neuronal hippocampal cultures and other neuronal systems [115,116]. Bicyclic monoterpene structures, such as borneol and its enantiomer, have also been shown to exert positive modulatory effects at GABA_A_ receptors [117].

### 2.3. Anthriscus nemorosa (Beaked/Rough Chervil, Beaked Parsley)

*A. nemorosa* (Bieb) Sprengel is a nitrophile perennial occurring in the temperate regions of Eurasia, from Italy in the West to Japan in the East [118]. It is an aromatic member of the Apicaceae family used traditionally around the world for medicinal purposes; the fruits of the plant are used to treat gastrointestinal ailments, inflammation, and rheumatism, and its essential oil is purported to improve memory [51]. The neuropharmacological effects of inhaled *A. nemorosa* essential oil in scopolamine-treated rats was investigated by Bagci et al. [51]. Scopolamine is a high-affinity muscarinic receptor antagonist that induces cognitive dysfunction by crossing the blood brain barrier in the CNS causing a cholinergic deficit, impairing memory [119]. *A. nemorosa* essential oil administered via inhalation at 1% and 3% concentration significantly reduced anxiety-like behaviour in scopolamine-treated rats in the elevated plus maze compared to the scopolamine-alone treated rats, with a 3% concentration demonstrating the most notable effects [51]. The sensitivity in mice to *A. nemorosa* essential oil in the elevated plus maze plus diazepam challenge suggest it activates the GABA_A_ receptor complex. The use of diazepam as a positive control in the experiment supports this, as it is known to enhance GABA binding to GABA_A_-benzodiazepine receptors [51]. 

### 2.4. Aquilaria spp. (Agarwood)

Agarwood is a resin-infused fragrant wood derived from the *Aquilaria, Gyrinops*, *Aetoxylon*, and *Gonystylis genera* [120]. The resin is formed in response to infection and/or internal injury in the stems of the agarwood tree. It is highly revered in seminal texts of Buddhism, Christianity, Hinduism, and Islam, and in 65 B.C.E. a detailed account of several medical applications was recorded by Dioscorides [120]. Agarwood has been a part of Traditional Chinese and Ayurvedic medicine for centuries and commonly used to treat inflammatory-related disorders and joint pain, as well as a sedative and cardioprotective agent [121]. It is an essential ingredient in oriental incense and sachets, and in Europe and the Middle East, the oil is commonly used in perfume and balm [52]. Reports have shown central nervous system effects of agarwood when administered orally and via injection. Takemoto et al. [52] investigated the sedative effects of agarwood oil and its principle constituents (benzylacetone, calaene, and α-gurjunene) administered by vapor inhalation in mice. Spontaneous motor activity was examined using an open field test. Vapor inhalation of agarwood oil and its principal constituents significantly reduced spontaneous locomotor activity in mice compared to control. Based on these findings, the authors imply that agarwood oil and its principal constituents exhibit sedative activity and may show promise in treating nervous system disorders. The reduced locomotion in the open field test, however, may not necessarily reflect sedative effects as other measures of motor activity including fear (anxiety) and exploratory drive (curiosity) can influence movement in the open field [25]. Freezing, motor output, relative time in circadian cycle, sickness, and a variety of other factors can also influence movement in the open field [25]. Although, to date, studies have not shown that the aroma of agarwood oil exerts a direct effect via the GABAergic system, Wang et al. [122] have confirmed oral administration of agarwood essential oil in mice to exert its sedative-hypnotic effects via potentiating GABA_A_ receptor function and regulating GABA_A_ receptor gene expression. GABA_A_ receptor function was measured via intracellular chloride concentration and gene expression via a real-time polymerase chain reaction investigating the mRNA level of GABA_A_ receptor subunits and subtypes in the cerebral cortex [122]. 

### 2.5. Cananga odorata (Ylang-Ylang)

*C. odorata* is a perennial tropical tree that grows natively in South-East Asian countries, known for its weeping branches and is well known in India, Philippines, and Malaysia, and the Pacific including the Pacific islands and Australia [123]. Ylang-ylang has known aphrodisiac, antidepressant, and sedative properties and was used in the Victorian age to encourage hair growth, to sooth insect bites, and to regulate respiratory and cardiac rhythms [3] (p. 203). Zhang et al. [53] evaluated *C. odorata* essential oil for its anxiolytic effects in male and female mice. To elucidate the anxiolytic potential of inhaled *C. odorata* essential oil, the researchers employed three anxiolytic models. Acute and chronic exposure of *C. odorata* essential oil resulted in significant anxiolytic effects in male mice observed in the elevated plus maze and light-dark box test. Anxiolytic effects were not observed in female mice. It is unclear why; although often ascribed to gonadal hormones, it is plausible that the different compounds in *C. odorata* act on multiple pathways as the monoterpenoids geraniol and nerol have shown potent estrogenic effects, while the phenylpropanoid eugenol has shown anti-estrogenic activity [53]. The authors also investigated the chemical isolates linalool, benzyl benzoate, and benzyl alcohol from *C. odorata* essential oil for their anxiolytic potential. Positive findings were found for all three chemical isolates in the elevated plus maze compared to control, however, only benzyl alcohol showed positive effects in the light-dark box test compared to control. These results suggest that the anxiolytic effects of *C. odorata* essential oil may work via synergism among the constituents. Although the mechanism of how *C. odorata* works is yet to be elucidated, the vapour from the monoterpenoid linalool, one of its main components, significantly potentiated GABAergic currents and modulated GABA_A_ receptors in human embryonic kidney cells (HEK293 cells) and *Xenopus* oocytes [124], suggesting a potential mechanism of action.

### 2.6. Chamaecyparis obtusa

In Japan, relaxing effects from the essential oil of *C. obtuse* (from a species of cypress native to central Japan in East Asia) have been reported [54]. *C. obtuse* essential oil was investigated in mice for its effect on anxiety and stress. Mice exposed to *C. obtuse* essential oil inhalation (7.0 mg/L air) displayed anxiolytic-like effects versus control in the elevated plus maze [54]. It is important to point out that *C. obtuse* essential oil contains large amounts of the monoterpene hydrocarbon α-pinene and is known for its anxiolytic effects via inhalation [125]. *A zerumbet* also contains α-pinene as one of its main components, and positive anxiolytic effects were observed in mice following inhalation [114]. Thus, the authors suggest that the anxiolytic-like effects are partly attributed to α-pinene. α-pinene has been shown in mice to prolong GABAergic synaptic transmission, is a partial modulator of GABA_A_-benzodiazepine receptors, and has capacity to bind to the benzodiazepine binding site of GABA_A_ receptors [126]. Stress-induced biomarkers (nerve growth factor receptor (NGFR), brain-derived neurotrophic factor (BDNF), galactokinase 1 (GLK1) protein expression, and activity-regulated cytoskeletal-associated protein (Arc) gene expression) within the brain were also examined after the elevated plus maze in the experiments by Kasuya et al. [54]. Results showed a significant increase in nerve growth factor receptor and Arc gene expression activity after inhalation of *C. obtuse* essential oil, indicating stress mitigation effects. Findings confirm that *C. obtuse* essential oil has both anxiety and stress-reducing effects. 

### 2.7. Citrus bergamia

*C. bergamia* is also known as “Bergamot” and belongs to the Rutaceae family, defined as a hybrid of bitter orange and lemon [127]. Native to tropical Asia and extensively cultivated in southern Italy it grows to 4.5 metres high with smooth oval leaves, bearing small round fruit [3] (p. 53). The oil has been used primarily for fever and worms in Italian folk medicine and is more recently used for infections, anxiety, and stress-related conditions [3] (pp. 53–54). Saiyudthong and Marsden [61] investigated the effects of *C. bergamia* oil inhalation on stress and anxiety in male rats. Rats exposed to *C. bergamia* oil inhalation at concentrations of 1% and 2.5% increased the open-arm percentage of entries into the elevated plus maze and at 2.5%, and 5% increased the percentage of time spent in the open arms. At 2.5% concentration, a significant increase in the number of head dips in the hole-board test and an attenuation of plasma corticosterone response to acute stress was observed. Findings confirm that *C. bergamia* oil administered via inhalation has both stress and anxiety reducing effects. Interestingly, bergamot essential oil contains unidentified monoterpene hydrocarbons that are able to stimulate glutamate and GABA release in hippocampal rat synaptosomes [128]. Equilibrium between glutamate and GABA is crucial for the proper functioning of the CNS and in anxiety disorders, there is a loss of this equilibrium with increased glutamate levels [129]. Bergamot essential oil may be able to correct this equilibrium providing anti-anxiety effects. It should be noted that bergamot essential oil was focally administered via a microdialysis probe, whereas systemic administration did not yield the same results [128]. It is unknown whether inhalation would have the same effect. 

### 2.8. Citrus limon (Lemon)

In herbal folk tradition, lemon is considered a ‘cure-all’ especially in relation to infectious diseases and when taken internally; the juice is beneficial for dysentery, arthritis, and rheumatism [3] (p. 122). Recently, lemon essential oil has been shown to improve mood and cognition and prevent cognitive decline during test anxiety. A randomised pretest-posttest design assessed lemon essential oil aroma on cognitive test anxiety in 39 nursing students [65]. Results confirmed diffused lemon essential oil decreased cognitive test anxiety scores in students who received the aroma compared to those who did not. Thus, diffused lemon essential oil had a positive effect on cognitive test anxiety among nursing students and was considered a safe and cost-effective intervention [65]. The same author evaluated cognitive test anxiety scores among 31 nursing students using inhaled lemon essential oil using the same pretest-posttest design with a different deliver system of the essential oil using a personal hand-held nasal inhaler, similar to a Vick’s inhaler [66]. The results did not produce a statistical difference in decreasing cognitive test anxiety scores following the lemon essential oil intervention [66]. The mode of delivery of the essential oil and duration could account for the difference in result. Exposure to the diffused lemon essential oil was continual during the entire 25-min duration of the examination whereas a personalised inhaler was applied when feeling any anxiety symptoms during the 75-min examination. The latter demonstrated more inconsistent exposure. 

The effect of lemon odour on anxiety was examined in female and male rats. Anxiety was measured using an elevated plus maze test. Lemon essential oil was administered in the rat’s cage preceding and during the behavioural test. Both sexes of rats exposed to lemon essential oil significantly decreased the percentage of time spent in the open arms. These findings reveal that long-term olfactory exposure to lemon odour increased anxiety in rats [64]. Contrary to these findings, Komiya et al. [62] examined the anti-stress effects of lemon oil vapour in mice using behavioural analysis and found anxiolytic effects in both the elevated plus maze and open field test following lemon oil inhalation compared to mice in other groups. These results show that lemon oil vapour exerts anti-stress and anxiolytic effects in distressed mice. 

The neuropharmacology of inhaled lemon oil extracts has suggested a potentiation of serotonergic transmission via a 5HT1a receptor interaction [62]. In other herbal Chinese medicines such as Gan-Mai-Da-Zao, both 5-HT_1A_ and GABA_A_ receptors are involved in translational models of anxiety and stress when taken orally in mice [130]. It is yet to be determined whether these effects would be similar via inhalation. 

### 2.9. Citrus spp. (Orange/Sweet Orange)

*Citrus* essential oils have known sedative effects and have become popular for treating anxiety and depression [55]. *Citrus aurantium* L. (orange) is an aromatic plant considered a popular alternative medicine for treating anxiety. Leite et al. [56] investigated the inhalation of orange essential oil for its anti-anxiety effect on rats via experimental models of anxiety. Inhalation of orange essential oil at a concentration of 2.5% increased both the time animals spent in the open arms of the elevated plus maze and time of active social interaction in the social interaction test. The open field test showed no significant changes except a decrease in faecal droppings compared to control. Decreased faecal droppings may correlate with a low level of emotionality as anxiogenic situations present with a larger number of faecal droppings [56]. Results from both experimental models of anxiety suggest inhalation of orange essential oil exhibits anti-anxiety activity [56]. 

Behavioural tests in rats have also been previously performed by Faturi et al. [67] where the anxiolytic effects of *C. sinensis* essential oil (sweet orange) aroma was investigated. Rats were subject to behavioural tests whilst exposed to different doses (100 µL, 200 µL, 400 µL) of sweet orange aroma. All doses of *C. sinensis* essential oil demonstrated anxiolytic activity in at least one of the tests, however the highest dose increased exploration in the open arms of the elevated plus maze and in the lit chamber of the light-dark box test. The researchers concluded that inhalation of sweet orange essential oil possesses acute anxiolytic activity. Wolffenbüttel et al. [55] evaluated the anxiolytic effects of *C. sinensis* and *C. aurantium* essential oil aromas via behavioural testing using the light-dark box test in mice. Findings revealed anxiolytic-like and sedative effects from inhalation of 10% of *C. sinensis* essential oil, however inhalation of 10% *C. aurantium* did not yield the same result. Thus, *C. sinensis* essential oil is considered more specific for anxiety reduction. Both *C. sinensis* and *C. aurantium* essential oil contain high amounts of limonene and, when administered intraperitoneally in mice, it inhibits anxiety-related behaviour via modulation of adenosine A2A receptor activity regulating GABAergic and DAergic neuronal activity [131]. Costa et al. [132], however, report that mice participated in serotonergic neurotransmission but not GABAergic/benzodiazepine neurotransmission following oral administration of *C. aurantium* essential oil. In the light-dark box procedure, anxiolytic-like effects were not antagonized by flumazenil, a benzodiazepine antagonist, yet these effects were antagonized by the 5-HT1A specific antagonist WAY100635, confirming mediation by the serotonergic system (5-HT1A receptors). 

### 2.10. Human Examples of Orange Essential Oil in Reducing Stress and Anxiety

Hasheminia et al. [57] conducted a randomised clinical trial (*n* = 56) where the fragrance from orange essential oil was evaluated for its ability to reduce anxiety before and during surgical removal of an impacted mandibular third molar. Dental anxiety scale (DAS) was employed to assess anxiety before surgery, and only participants with moderate and high anxiety levels on the scale were included. Physiological measures relating to anxiety, including mean blood pressure, respiratory rate, and pulse rate, were recorded before and after the surgery. Results indicated that participants exposed to the orange fragrance had significantly lower mean blood pressure, respiratory rate, and pulse rate. Thus, orange fragrance is effective at reducing anxiety preoperatively [57]. Another randomised clinical study (*n* = 42) assessed the anxiolytic activity of inhaled orange essential oil in chronic myeloid leukemia (CML) patients. Evaluation of anxiety was performed using the STAI and physiological measures, including blood pressure, cardiac, and respiratory frequencies. A significant decrease in STAI-State scores and all physiological measures was observed in the orange essential oil group. Findings from the study demonstrate orange essential oil to reduce the signs and symptoms associated with anxiety in patients with Chronic Myeloid Leukemia [58]. 

Namazi et al. [59] evaluated the effects of inhaled orange essential oil on anxiety in pregnant women in their first stage of labour. A total of 126 primiparous women were randomly assigned into either an aromatherapy (*n* = 63) or control (*n* = 33) group. Anxiety was measured via the Spielberger state-trait anxiety questionnaire. Levels of anxiety were significantly lower in the aromatherapy group at dilations of 3–4 and 6–8 cm compared to the control group. This study confirmed that orange essential oil is an effective intervention to reduce anxiety during labour. 

Inhalation of orange essential oil (two drops) was evaluated in withdrawing drug crack cocaine (a free base form of cocaine) users in a randomised clinical trial (*n* = 51) for its effect on anxiety. Individuals experiencing crack cocaine withdrawal present with high anxiety traits. The Simulated Public Speaking method was used to induce anxiety and psychological (STAI and HAS) and physiological (end temperature, skin electric conductance, systolic, and diastolic blood pressure and heart rate) measures of anxiety were assessed [38]. Heart rate variability is reflective of the cardiac autonomic nervous system (ANS) and can be used to assess acute stress responses via the ANS and gauge the interaction between the sympathetic and parasympathetic nervous systems [133]. Orange essential oil was efficient in controlling the psychological parameters but not the physiological parameters during the anxiogenic task. Results from the study demonstrated that nebulization of orange essential oil is an acute anti-anxiety treatment for withdrawing crack cocaine users subject to a simulated Public Speaking task [38].

Recently a double-blind placebo-controlled trial (*n* = 140) investigated the aroma of orange essential oil for anxiety in patients with acute coronary syndrome (ACS) using the STAI as an anxiety measure. Findings from the study revealed a significant reduction in anxiety scores in the orange essential oil group compared to placebo. Thus, the aroma from orange essential oil is considered a safe and efficient intervention for treating anxiety in patients with ACS [60]. 

Clinical studies have revealed anxiolytic properties of *C. sinensis* oil aroma. The ambient odour of sweet orange essential oil was evaluated in 72 patients waiting for dental treatment. A lower level of state anxiety, a higher level of calmness, and a more positive mood was reported in women exposed to orange odour compared to controls [68]. Pour et al. [69] investigated the effect of the aroma of *C. sinensis* essential oil on child anxiety during dental treatment in a randomised controlled clinical trial. Results from the study revealed a significant decrease in salivary cortisol and pulse rate in children exposed to the aroma of *C. sinensis* essential oil compared to controls. These findings suggest that the aroma of *C. sinensis* essential oil has anxiolytic potential in children during dental treatment. Goes et al. [70] evaluated the anxiolytic potential of *C. sinensis* aroma in 40 healthy male volunteers subjected to an anxiogenic task—a video-recorded Stroop Color-Word Test (SCWT). The Stroop test represents the ability to inhibit cognitive interference, and assesses the delay in reaction time between congruent and incongruent colour word stimuli [134]. It is a simple method that uses subject scales to predict clinical activity of anxiolytic drugs [135]. Individuals exposed to *C. sinensis* aroma exhibited a lack of significant alterations in state-anxiety, tranquility levels, and subject tension throughout the SCWT as per the STAI and VAMS, revealing anxiolytic activity of sweet orange essential oil. The present results indicate acute anxiolytic effects of sweet orange aroma. 

### 2.11. Coffea spp. (Coffee)

Coffee is a popular beverage consumed worldwide, and numerous studies have confirmed beneficial effects on human health. Coffee aroma has been shown to induce relaxing effects on humans whilst restoring alertness. Koga [71] investigated the relaxation effects of six different types of coffee bean aromas on 10 healthy, right-handed women measuring alpha waves from electroencephalographic recordings. Alpha waves activity significantly increased with exposure to Guatemala compared to Mandheling or Hawaii Kona blends of coffee beans. These findings indicate that the relaxing effect of coffee is dependent on bean type. Hayashi et al. [136] examined the anxiolytic effects of roasted Guatemala coffee bean volatile compounds in mice using a variety of behavioural tests. The number of open-arm entries and time spent in the open arms of the elevated plus maze increased in a dose-dependent manner following exposure to coffee volatiles, and locomotor activity remained unchanged in the open field test. These results imply that coffee volatiles exert moderate anxiolytic effects without affecting normal motor activity. Although a direct effect on GABA_A_ receptors is yet to be shown from the aroma of coffee, Hossain et al. [137] demonstrated that an aqueous coffee extract and coffee components elicited a GABA_A_ receptor response in *Xenopus* oocytes. 

### 2.12. Compound Anshen

Compound Anshen is a blend of essential oils based on the theory of aromatherapy of Traditional Chinese Medicine, consisting of lavender, sweet orange, sandalwood, frankincense, orange blossom, rose, and agarwood oils. Zhong et al. [72] investigated the sedative effects of compound Anshen essential oil inhalation in mice. Results from the open field test indicate that mice who inhaled compound Anshen essential oil had a significant reduction in spontaneous activity (rest time, distance moved, average and maximum velocity, and number of arm lifting). Based on these findings, the authors imply that compound Anshen exhibits sedative effects [72]. The reduced locomotion in the open field test may, however, not necessarily reflect sedative effects as other measures of motor activity including fear (anxiety) and exploratory drive (curiosity) can influence movement in the open field [25]. It has been suggested that the sedative properties from compound Anshen are due to its main active constituents (linalool, caryophyllene, dibutyl phthalate, (-)-4-terpineol, and (-)-α-terpineol) [138]. Predictive pharmacology analysis indicates that the active constituents in compound Anshen have sedative and anxiolytic effects. It is also worthwhile knowing that the study examined brain neurotransmitters, and there was a significant increase in 5-HT and GABA in mice exposed to compound Anshen following the open field test. The active constituent’s linalool and terpinen-4-ol have previously shown GABAergic neurotransmission involvement. Linalool vapour significantly potentiated GABAergic currents and modulated GABA_A_ receptors in human embryonic kidney cells (HEK293 cells) and *Xenopus* oocytes [124]. Terpinen-4-ol inhibited 3-MPA, a glutamic acid decarboxylase inhibitor, induced convulsions but did not reverse flumazenil, a selective antagonist of the benzodiazepine-GABA_A_ receptor site, confirming that the action of terpinen-4-ol is directly or indirectly related to the GABAergic system but does not act on the GABA_A_ receptor benzodiazepine site [44].

### 2.13. Coriander sativium (Coriander)

*C. sativium* is an aromatic, herbaceous annual plant belonging to the umbelliferae/apiaceae and widely used in folk medicine and in culinary preparations as a seasoning agent. The essential oil form *C. sativium* has a long history of use as a traditional medicine and a decoction and tincture of the powdered seeds of *C. sativium* alone or in combination with other herbal agents has been used for a loss of appetite, dyspesia, convulsions, insomnia, and anxiety [139]. Cioanca et al. [20] investigated the anxiolytic effects of the inhalation of *C. sativum* essential oil in rats. Rats were injected with beta-amyloid (1–42) to replicate a dementia model and exposed to an elevated plus maze test. Entries and percentage of time spent in the open arms were significantly reduced in rats with the pathological brain Aβ (1–42), indicating that these rats experienced high levels of anxiety. The opposite was found in the elevated plus maze when rats were exposed to the aroma of *C. sativum* essential oil in a dose-dependent manner indicating anxiolytic effects. In addition, the locomotor activity of rats treated with inhalation of *C. sativum* essential oil also increased [20]. *C. sativum* contains a significant amount of the monoterpene hydrocarbon α-pinene and the monoterpene alcohol linalool [20,102], which may be responsible for the anxiolytic effects. Previous studies have shown that plants high in α-pinene, such as *A. zerumbet* and *C. obtusa,* have shown positive anxiolytic effects via inhalation [54,114] and Satou et al. [125] have confirmed the anxiolytic effects of α-pinene when inhaled. α-pinene is known to prolong GABAergic synaptic transmission, partially modulate GABA_A_-benzodiazepine receptors, and bind to the benzodiazepine binding site of GABA_A_ receptors [126]. Linalool vapour derived from the *Sideritis* species significantly potentiated GABAergic currents and modulated GABA_A_ receptors in human embryonic kidney cells (HEK293 cells) and *Xenopus* oocytes [124]. 

### 2.14. Cryptomeria japonica (Japanese Cedar)

*C. japonica*, known as “sugi” in Japanese, is a very large evergreen conifer belonging to the Cupressaceae family. The wood is reddish-pink in colour, scented, water proof, resistant to decay, and lightweight but strong [140]. The essential oil from *C. japonica* is predominately derived from the foliage, although it can be derived from the wood and roots, and possesses a range of medicinal properties, including antimicrobial activities; it is also used in art, perfumery, and in aromatherapy to renew the smell of natural cedar furniture and as an insect repellent [140]. In Japan, *C. japonica* is commonly planted in forests and the timber is used as building material for interior walls [73]. Matsubara and Kawai [73] examined the volatile organic compounds (VOCs) emitted from the interior walls made of Japanese cedar on psychophysiological responses in a crossover design study. A total of 16 healthy males aged between 21 and 28 were placed in an experimental room containing Japanese cedar wood and subjected to an arithmetic task (Uchida-Kraepelin test—a serial addition test calculating as quickly and accurately as possible 15 lines with single digits aligned horizontally in random order in repeated 15-min cycles with five minutes rest). Japanese cedar VOCs suppressed the increase in salivary stress markers (α-amylase and chromogranin A) and only in the control group there was an increase in low-frequency to high-frequency ratio of the ECG. Thus, VOCs emitted from Japanese cedar suppressed the activation of the sympathetic nervous system during and after the arithmetic task inducing physiological relaxation. In line with these findings, Matsubara and Ohira [74] found that inhalation of Japanese cedar essential oil decreased sympathetic nervous system activity and induced positive mood-altering effects in 29 female participants after performing a laboratory arithmetic work task. Findings from both studies imply that Japanese cedar exhibits relaxation properties following a stress-induced work task. It is unknown whether *C. japonica* or its main compounds exert anxiolytic effects via the GABAergic system.

### 2.15. Cymbopogon citratus (Lemon Grass)

*C. citratus* belongs to the poaceae family and is known for its sweet, herbaceous, and lemony fragrance. Native to India, Pakistan, and Sri Lanka, it is widely used in herbal teas, non-alcoholic beverages, and confectionary. The essential oil from *C. citratus* is used medicinally for its anti-depressant, analgesic, antipyretic, anti-septic, and antibacterial activities, and its fragrance is commonly used in perfumery and cosmetics [141]. In tropical countries, *C. citratus* is widely used as a source of medicine, and in Brazil it is used medicinally as a tea for its analgesic, antipyretic, anti-inflammatory, and tranquillising properties [141]. It has been traditionally used as an infusion for treating nervous disturbances, and its essential oil has been shown to elicit anxiolytic effects following oral administration in mice [43]. The anxiolytic activity of *C. citratus* aroma was evaluated in 40 male healthy subjects between the ages of 18 and 30. To elicit anxiety, a SCWT was performed. Psychological parameters of state anxiety, subjective tension, tranquilisation, and sedation (assessed via the STAI and VAMS) and physiological (heart rate and gastrocnemius electromyography activity) parameters were measured before the inhalation period and before, during, and after the SCWT [75]. *C. citratus* aroma at both concentrations (three and six drops) was unable to prevent the increase in anxiety caused by the SCWT, however subjective tension and basal levels of anxiety were reduced immediately after administration with the lemon grass aroma compared to controls. These results were unexpected as other researchers have observed that diazepam was not able to reduce the basal levels of anxiety in the SCWT in healthy volunteers [135]. In addition, subjects treated with *C. citratus* aroma (six drops) recovered from the anxiogenic task within five minutes, unlike the control groups. Thus, brief exposure to *C. citratus* aroma has perceived anxiolytic effects, although further investigations are needed to clarify its clinical relevance [75]. Costa et al. [43] found in mice that *C. citratus* essential oil taken orally at a dose of 10 mg/kg produced anxiolytic effects in the light-dark box test and that these anxiolytic effects occur through the GABAergic system as evident by a reversal of the anxiolytic-like activity of the essential oil when co-administered with flumazenil, a benzodiazepine GABA_A_ site antagonist. Furthermore, Silva et al. [142] have shown *C. citratus* essential oil to have an anticonvulsant effect, which was blocked by flumazenil, indicating GABAergic system involvement. 

### 2.16. Eucalyptus globulus

*E. globulus* is a broad-leaf evergreen flowering tree belonging to the Myrtaceae family and native to Australia, Africa, and South America. It has been used for thousands of years throughout human history and is therapeutically highly valuable possessing anti-microbial, anti-inflammatory, analgesic, anti-nociceptive, and antioxidant properties [143]. In a quasi-randomized controlled pilot study in 123 cancer patients scheduled to undergo chemotherapy, Yayla and Ozdemir [76] examined the effect of *E. globulus* inhalation on procedural pain and anxiety after needle insertion into an implantable venous port catheter. Anxiety was measured using the STAI. There was no significant difference in average STAI scores following *E. globulus* inhalation. In another randomized controlled trial (*n* = 62) inhalation of eucalyptus oil and its components were assessed for their anxiolytic effects on patients prior to a non-surgical treatment (Sensory Nerve Root Block (SNRB)) [32]. Preoperative anxiety was measured using STAI, POMS, and A-VAS. Patients were assigned into four groups of >15 with three treatment groups (eucalyptus, 1,8-cineole, limonene) and one control group (almond oil). Anxiety measures (STAI, POMS, A-VAS) were significantly lower in all groups, with the 1,8-cineole group being significantly lower than the control group [32]. 

1,8-cineole is the major active constituent of eucalyptus and its inhalation is effective at reducing preoperative anxiety in patients prior to SNRB; it may be a useful therapeutic for relieving preoperative anxiety in other operations [32]. Mice studies performed by Dougnon and Ito [144] demonstrated anxiolytic-like effects from the inhalation of 1,8-cineole as evident by increased number of entries and time spent in the light box of the light-dark box test and reduced number of marbles buried compared to control in the marble-burying test. In addition, the researchers investigated the GABA_A_ benzodiazepine receptor system by administering a GABAergic system antagonist (flumazenil) which reversed the effects of 1,8-cineole suggestive of an effect on the GABA_A_ benzodiazepine receptors. 

### 2.17. Forest Abies sachalinensis

*A*. *sachalinensis* is a representative evergreen conifer species of northern Japan [145]. The volatile components obtained from the needles of the *Abies* genus have been utilised in *Shirin-yoku*, a practice of forest bathing in Japan as well in aromatherapy for their relaxing and anti-microbial properties [77]. To determine the anxiolytic effects of the *A. sachalinensis* essential oil, Satou et al. [77] experimented on mice using an elevated plus maze task. Mice exposed to *A. sachalinensis* essential oil via inhalation at concentrations of 2.7 mg/L and 3.6 mg/L spent significantly more time in the open arms, implying anxiolytic effects. Surprisingly, intraperitoneal (i.p.) administration of the essential oil at all concentrations (0.3 g/kg, 0.45 g/kg and 0.6 g/kg) did not yield anxiolytic effects, indicating that brain levels and other factors, such as olfactory sense, are important elements in mediating the anxiolytic-like effects of *A. sachalinensis* essential oil. In addition, brain levels of the essential oil components appear to be proportionally higher when inhaled compared to other routes of administration [77]. The efficacy of *A. sachalinensis* essential oil could be due to achieving adequate levels in the brain following inhalation [77]. *A. sachalinensis* essential oil is rich in the monoterpene hydrocarbons α-pinene and camphene, and reports have shown anxiolytic-like effects for both components [114,125,146,147]. Whether *A. sachalinensis* exerts its anxiolytic effects through the GABAergic system is unknown; however, one of its main components α-pinene has shown benzodiazepine receptor involvement [146], and Yang et al. [126] have confirmed anxiolytic effects via prolonged GABAergic synaptic transmission, partial modulation of GABA_A_-benzodiazepine receptors, and binding to benzodiazepine binding site of GABA_A_ receptors, suggesting a possible mechanism of action. 

### 2.18. Fragrant Compounds from Oolong Tea

Oolong tea is partially fermented, giving it a distinct fruity, floral, and jasmine-like aroma. Various pharmacological properties have been attributed to oolong tea, including antioxidant, anti-obesity, anti-diabetic, anti-cancer, anti-allergic, and preventative effects against atherosclerosis, heart disease, and hypertension [148]. In addition, stress-reducing effects have been shown from consumed oolong tea and GABA-fortified oolong tea [149]. Fragrant compounds in oolong tea were investigated for their response on ionotropic GABA_A_ receptors in xenopus oocytes [78]. *Cis*-jasmone, methyl jasmonate, jasmine lactone, and linalool oxide all significantly potentiated the GABA_A_ receptor response with *Cis*-jasmone and methyl jasmonate potently potentiated the response. In addition, sleeping time of mice induced with pentobarbital (known to potentiate the GABA_A_ receptor response) and exposed to inhalation of *Cis*-jasmone and methyl jasmonate was examined. Results showed that *Cis*-jasmone and methyl jasmonate significantly increased sleeping time in mice, implying that these fragrant compounds were absorbed by the brain, inducing a tranquillising effect via potentiating a GABA_A_ receptor response [78]. 

### 2.19. Heracleum afghanicum

*H. afghanicum* is a perennial plant indigenous to Afghanistan and the seeds of the plant have been used as a spice and its leaves used to treat pain and fevers [79]. Karimi and Ito [79] were the first to investigate the sedative effects of the essential oil from the seeds of *H. afghanicum* in mice. Vapour inhalation of *H. afghanicum* essential oil and its principal constituent’s hexyl butyrate and octyl acetate significantly decreased locomotor activity in the open field test compared to controls. The authors imply that hexyl butyrate and octyl acetate are responsible for the sedative effects in *H. afghanicum* essential oil. The reduced locomotion in the open field test, however, may not necessarily reflect sedative effects as other measures of motor activity including fear (anxiety) and exploratory drive (curiosity) can influence movement in the open field [25]. It is unknown whether *H. afghanicum* or its main compounds exert sedative effects via the GABAergic system. 

### 2.20. Hypericum scabrum

Members of the *Hypericum* genus have traditionally been used for a range of ailments, including mild to moderate depression. *Hypericum perforatum* (St John’s Wort) is the most well-known of the genus and has been investigated and used for its antidepressant effects [80]. Other species of *Hypericum,* such as *Hypericum scabrum,* have also been evaluated and have shown to possess a range of pharmacological actions, including sedative effects [80]. Recently, the essential oil was evaluated for its neuropharmacologoical effects in a dementia animal model. *H. scabrum* essential oil administered via inhalation at 1% and 3% concentration reduced anxiety-like behaviour in scopolamine-induced rats in the elevated plus maze with the 3% concentration significantly increasing the effect compared to the 1%. These results suggest that *H. scabrum* essential oil could be used as a therapeutic agent to reduce anxiety-like symptoms in patients with dementia [80]. The essential oil from *H. scabrum* contains a high amount of α-pinene that the authors believe attribute to its anxiolytic effects. Previous reports have shown α-pinene to prolong GABAergic synaptic transmission and to modulate and bind to GABA_A_-benzodiazepine receptors [126].

### 2.21. Illicium verum (Star Anise)

*I. verum*, commonly known as star anise is an evergreen tree belonging to the Lillaceae family and native to China and Vietnam, where it has been used for over 3000 years [150]. It has a long history of use in the food industry and in traditional medicine for preventing colds and relieving pain. The aromatic odour is due to the essential oil, which is chiefly comprised of trans-anethol, known for its antiviral and cancer-preventing activities. Pharmacology studies have demonstrated that its active constituents possess a broad range of properties, including antioxidant, anti-inflammatory, cytotoxic, antimicrobial, and sedative activities [150]. A previous in-vivo study demonstrated that polar and non-polar extracts (methanol, *n*-hexane, and ethyl acetate) of *I. verum* administered intraperitoneally (200 mg) in rats had potent CNS depressant effects by increasing phenobarbitone induced sleeping time and anxiolytic effects via increased entries and time spent in the elevated plus maze without interfering with motor coordination [151]. The fragrance of *I. verum* essential oil was investigated for its anxiolytic effects in mice via an elevated plus maze task. Results demonstrated that *I. verum* essential oil did not possess anxiolytic effects; however, the main component trans-anethole did [81]. It is unknown whether *I. verum* or its main components exert anxiolytic effects via the GABAergic system. 

### 2.22. Jasminum grandiflorum (Jasmine)

*J. grandiflorum* Linn. is a large evergreen shrub native to Asia, Kashmir, Afghanistan, and Persia and used as a popular remedy for a variety of uses recommended by Asian and Indian folk practitioners for liver complaints, menstrual pain, and skin disease such as leprosy [152]. In addition, jasmine oil has been applied externally to soften and smooth skin, for cancer, heart disease, and variety of other ailments, and aromatherapists believe it to be a useful calming agent to sooth stress and to relieve anxiety [152]. Kuo [82] investigated the effects *J. grandiflorum* essential oil inhalation has on the central nervous system in humans (*n* = 31) using a multi-functional physiological recorder. Findings revealed inhalation of jasmine essential oil increased peripheral blood flow and skin temperature and reduced respiratory frequency suggestive of a relaxation effect. In addition, *J. grandiflorum* essential oil significantly decreased heart rate variability parameters related to sympathetic nerves. Phytol and linalool are two significant constituents in *J. grandiflorum* that have shown to exert their influence via GABAergic neurotransmission. Phytol, a branched chain unsaturated alcohol, when acutely administered (i.p.) exerts anxiolytic-like effects that possibly interact with GABA_A_ receptors [153], and linalool vapour significantly potentiated GABAergic currents and modulated GABA_A_ receptors in human embryonic kidney cells (HEK293 cells) and *Xenopus* oocytes [124]. 

### 2.23. Lantana camara

*L. camara*, also known as red or wild sage, is an evergreen, strong-smelling shrub belonging to the Verbenaceae family and is a widespread species of the *Lantana* genus reported to be used in traditional medicine for the treatment of swellings, ulcers, cuts, itches, eczema, and rheumatism [154]. The leaves have been traditionally used in the Republic of Benin to treat skin ailments, and the essential oil from *L. camara* is purported to possess sedative effects when inhaled [84]. The sedative effects of *L. camara* essential oil inhalation were investigated in mice using an open-field test [84]. Results from the study revealed that mice exposed to *L. camara* essential oil at all doses significantly reduced locomotor activity in a dose-dependent manner compared to the control. Based on these findings, the authors suggest that *L. camara* essential oil administered as an inhalant possesses sedative activity. The reduced locomotion in the open field test, however, may not necessarily reflect sedative effects as other measures of motor activity, including fear (anxiety) and exploratory drive (curiosity), can influence movement in the open field [25]. Dougnon and Ito also investigated the chemical isolates from *L. camara* essential oil and described their influence on locomotor activity in an open field test. Mice treated with 1,8-cineole and sabinene via inhalation reduced locomotor activity compared to control, inferring a sedative effect. Inhalation of the naturally occurring monoterpene 1,8-cineole was shown to have anxiolytic effects in behavioural assessments of anxiety as measured by an increase in the amount of time spent and number of entries in the light-dark box as well as reduced number of marbles buried in the marble-burying test compared to controls [144]. Co-administration of 1,8-cineole and flumazenil, a GABAergic antagonist, reversed the effects of 1,8-cineole implying that 1,8-cineole affects the GABA_A_-benzodiazepine receptors. 

### 2.24. Lavandula spp. (Lavender)

The therapeutic use of lavender can be traced back to ancient Rome and Greece. The fragrance of lavender is known for its calming effects and has been traditionally used to induce sleep and in folk medicine to treat anxiety [85,86]. Inhalation of *Lavandula angustifolia* essential oil has shown promise in reducing anxiety with clinical and animal studies corroborating its anti-anxiety effect. Soni et al. [155] report in their review on the medical utility of lavender that *L. angustifolia* exerts a similar action to benzodiazepines and increases the effects of GABA in the amygdala. In addition, Aoshima and Hamamoto [88] have shown that the essential oil from *Lavandula officinalis* and *Lavandula hybrica* mildly potentiates GABA at GABA_A_ receptors. Contrary to these findings, Chioca et al. [85] demonstrated in mice that inhalation of *L. angustifolia* essential oil participated in serotonergic neurotransmission but not GABAergic/benzodiazepine neurotransmission. Mice pretreated with a GABA_A_ receptor antagonist and exposed to *L. angustifolia* essential oil had no effect on behavioural indices in the marble burying test; however, when mice were pretreated with a serotonin 5-HT_1A_ receptor antagonist, this blocked the anxiolytic effects of both *L. angustifolia* essential oil and a 5-HT_1A_ receptor agonist. Thus, the serotonergic system plays an important role in the anxiolytic effects of *L. angustifolia* essential oil. Vinkers and Oorschot [156] have shown that the α3-subunit of the GABA_A_ receptor functionally interacts with 5-HT_1A_ receptors of the serotonin system to exert anxiolytic effects. 

Recently, a randomized clinical trial evaluated *L. angustifolia* essential oil inhalation in children (*n* = 126) assigned to undergo tooth extraction using psychological (FBRS and FIS) and physiological (systolic and diastolic blood pressure, pulse/heart rate) measures. Inhalation of lavender essential oil resulted in lower anxiety and pain after tooth extraction as well as significantly lower blood pressure and pulse rate compared to control [87]. A systematic review and meta-analysis of lavender essential oil was conducted by Donelli et al. [157] and revealed lavender essential oil when administered via inhalation was effective in reducing anxiety and could be considered a clinical therapeutic option due to its simplicity, safety, and low cost. Considering the heterogeneity of the data and the high risk-of-bias of the trials, the authors recommend further high-quality RCTs to confirm such findings. 

Linalool and linalool acetate are major components present in lavender. Buchbauer et al. [158] investigated the effects of numerous fragrance compounds and essential oils in mice and found linalool and linalool acetate to demonstrate reductions in motility after one hour of inhalation. In addition, lavender oil, linalool, and linalool acetate were able to compensate for the caffeine-induced agitation. The results had been interpreted as lavender possessing sedative effects. Furthermore, linalool vapour significantly potentiated GABAergic currents and modulated GABA_A_ receptors in human embryonic kidney cells (HEK293 cells) and *Xenopus* oocytes [124]. Other components within the essential oil of *Lavandula* spp. have also shown GABAergic involvement with the monoterpene terpinen-4-ol directly or indirectly related to the GABAergic system but does not act on the GABA_A_ receptor benzodiazepine site [44] whereas the monoterpene borneol and is enantiomer exerted positive modulatory effects at GABA_A_ receptors [117].

### 2.25. Matricaria chamomilla

*M. chamomilla* is native to southern and eastern Europe and has been used in herbal remedies for thousands of years, known in ancient Greece, Egypt, and Rome. It has been used to treat flatulence, colic, intermittent fever, and hysteria, however it is mainly used as an anti-inflammatory, sudorific, antispasmodic, and antiseptic [89]. Yamada et al. [90] investigated inhalation of *M. chamomilla* essential oil in ovariectomised rats stressed by restrictive movement. There was a significant decrease in plasma adrenocorticotropic hormone in rats exposed to the vapour of *M. chamomilla* essential oil, indicating sedative effects. In addition, inhalation of *M. chamomilla* essential oil induced a greater reduction in plasma ACTH levels than ovariectomised rats treated with diazepam. In normal female rats, this difference was not observed. Furthermore, the decrease in plasma ACTH levels observed from *M. chamomilla* essential oil vapour was blocked by pre-treatment with flumazenil, a potent and specific benzodiazepine antagonist. These results imply *M. chamomilla* essential oil may effect GABAergic systems in the rat brain and may have similar activity to benzodiazepine agonists [90]. Tabari and Tehrani [159] report α-bisabolol, a main component in *M. chamomilla,* to have GABAergic system involvement when administered intraperitoneally. Mice treated with α-bisabolol spent more time in the open arms and increased the total number of entries in the elevated plus maze. In addition, the researchers demonstrated that the anxiolytic-like effects of α-bisabolol in the elevated plus maze were reversed with pre-treatment of flumazenil, a GABAergic antagonist, and not WAY-100635, a 5-HT1A specific antagonist, indicating α-bisabolol’s anxiolytic effects occur via GABAergic and not serotonergic transmission [159]. Other components within the essential oil of *M. chamomilla* have also shown GABAergic involvement with the monoterpene borneol and is enantiomer exerting positive modulatory effects at GABA_A_ receptors [117].

### 2.26. Microtoena patchouli (Patchoulii)

*M. patchouli* is a perennial plant belonging to the Lamiaceae family, grown in the highlands of southern China, northeastern India, and Myanmar. Its fresh leaves possess a strong aroma and have traditionally been used in folk medicine for asthma, coughs, abdominal pain, enteritis [160], and as an incense and perfume for its relaxing or energising effects [91]. The sedative activity of the essential oil of *M. patchouli* administered by inhalation has been examined in mice using an open field test. Mice treated with *M. patchouli* essential oil at a concentration of 0.4 mg and 4 mg significantly reduced spontaneous motor activity. Based on these findings the authors imply that inhaled *M. patchouli* essential oil at different concentrations may have induced sedative effects [91]. In addition to these findings, the chemical isolates 1-octen-3-ol, terpinolene and patchouli alcohol from *M. patchouli* essential oil showed a significant reduction in spontaneous motor activity at various concentrations. The reduced locomotion in the open field test, however, may not necessarily reflect sedative effects as other measures of motor activity including fear (anxiety) and exploratory drive (curiosity) can influence movement in the open field [25]. In other studies, compounds containing 1-octen-3-ol (100 mg/kg) orally administered to mice prior to intraperitoneal administration of pentobarbital increased the sleeping time associated with pentobarbital [137]. Further evidence of a direct effect of 1-octen-3-ol on significantly potentiating GABA-evoked currents in injected *Xenopus* oocytes and transfected HEK293 cells expressing GABA_A_ receptors has been shown [124].

### 2.27. Nardostachys chinensis (Spikenard)

*N. chinensis* is an aromatic used by the ancient Egyptians and is mentioned in the Song of Solomon in the bible as an herb that Mary used to anoint Jesus before the Last Supper [3]. Spikenard is found in the Himalayas of Nepal, China, and India and has traditionally been used as a sedative and tranquiliser. Takemoto et al. [52] investigated the sedative effects of *N. chinensis* administered by vapor inhalation in mice. Spontaneous motor activity was examined using an open field test. Vapor inhalation of *N. chinensis* significantly reduced spontaneous locomotor activity in mice compared to control. Based on these findings, the authors imply that *N. chinensis* exhibits sedative effects. The reduced locomotion in the open field test, however, may not necessarily reflect sedative effects as other measures of motor activity including fear (anxiety) and exploratory drive (curiosity) can influence movement in the open field [25]. Modern pharmacological studies have revealed that chemical isolates from *N. Chinensis* have sedative and anti-anxiety qualities when inhaled [161]. 

Inhalation of the chemical isolates patchouli alcohol and aristolen-1(10)-en-9-ol demonstrated sedative effects in caffeine-treated mice in the open field test [92]. In addition, aristolen-1(10)-en-9-ol prolonged the pentobarbital-induced sleep time in mice. This effect was completely reversed following administration of flumazenil, a GABA_A_-benzodiazepine receptor antagonist [92], inferring that the sedative effect is expressed via the GABAergic system. Furthermore, aristolen-1(10)-en-9-ol was comparable to diazepam for its sedative action but did not impair motor coordination [92]. The chemical isolate patchouli alcohol also demonstrated sedative effects in the open field test of caffeine-treated mice. 

### 2.28. Ocimum spp. (Basil)

*O. basilicum* (basil), also known as sweet basil, is a universally cultivated herbaceous perennial plant belonging to the Lamiaceae family and used as a culinary herb and medicinally to prevent or treat digestive, cardiovascular, and neurodegenerative disorders [162]. Extracts of the essential oil have been used to flavour food products, as a commercial fragrance, to improve shelf life of food products, and medicinally as an antimicrobial agent [162]. Hirai and Ito [93] investigated the sedative effects of the inhaled essential oil extracted from *O. basilicum* and the headspace air of this living plant in mice. The researchers concluded that both the inhaled essential oil extract and headspace air of *O. basilicum* demonstrated sedative properties in the open field test via reducing locomotor activity in mice. The reduced locomotion in the open field test, however, may not necessarily reflect sedative effects as other measures of motor activity including fear (anxiety) and exploratory drive (curiosity) can influence movement in the open field [25].

Previous research by Tankam and Ito [95] demonstrated that inhalation of *Ocimum gratissimum* (African Basil) essential oil exerted potent sedative and anxiolytic-like effects in behavioural tests in mice (open field and light-dark box tests) without any impairment to motor coordination. Findings from both studies suggest that basil may have aromatherapeutic potential for its sedative and anxiety reducing effects. In agreement with these studies, Gradinariu et al. [94] demonstrated anxiolytic effects of inhalation of *O. basilicum* and *Ocimum sanctum* essential oil’s in a dementia rat model as shown by a reduction in anxiety indices in the elevated plus maze test. 

Gradinariu et al. [94] identified linalool as the main compound in both *Ocimum basilicum* (31%) and *Ocimum sanctum* (19%), and Tankman and Ito [95] identified thymol (68%) as the main compound in *Ocimum gratissimum*. Linalool vapour has been shown to ellicit a GABAergic response in human embryonic kidney cells (HEK293 cells) and *Xenopus* oocytes via potentiating GABAergic currents and modulating GABA_A_ receptors [124]. Thymol has been shown to potentiate GABA_A_ receptors expressed in *Xenopus laevis* oocytes [163]. These findings suggest that linalool and thymol act directly or indirectly on GABAergic neurotransmission and could possibly explain a potential mechanism of action for the anxiolytic effects of *Ocimum* spp.

### 2.29. Pelargonium graveolens (geranium)

*P. graveolens*, commonly known as geranium, is a perennial plant that belongs to the Geraniaceae family, purported to originate in South Africa and introduced to Europe in the 17th century and is used in traditional medicine to treat malaria, respiratory tract infections, abdominal, uterine, and gastrointestinal disorders [164]. The essential oil is utilised in perfumery, cosmetics, and aromatherapy and has shown antimicrobial, antitumor, and immune modulating effects [164]. Its aroma has recognised sedative and anti-anxiety properties [6]. Recently geranium was clinically examined for its ability to reduce anxiety. In a triple-blind randomised clinical trial, inhalation of *P. graveolens* essential oil significantly reduced anxiety in patients with acute myocardial infarction as measured by the STAI [96]. In another randomised clinical study, inhalation of *P. graveolens* essential oil was effective at reducing anxiety scores in the STAI and reducing diastolic blood pressure (physiological measure) in 100 nulliparous women in the first stage of labour, confirming anti-anxiety effects [97]. *P. graveolens* essential oil contains large amounts of the acyclic monoterpene citronellol, which has been shown to potentiate GABA_A_ receptors expressed in *Xenopus oocytes* [88] confirming GABAergic system involvement and possibly explaining a potential mode of action for its anxiolytic effects. 

### 2.30. Phytoncides

Phytoncides are volatile or non-volatile compounds produced by plants that have an influence on another organism [165]. Forest trees and plants emanate volatile compounds such as α-pinene, *cis*-3-hexenol (leaf alcohol), and *β*-thujaplicin (hinokitiol), commonly known as phytoncides. It is known that walking in the forest with the scent of phytoncides induces anxioxlytic and sedative effects [165]. Aoshima and Hamamoto [88] studied the effects of phytoncides on GABA_A_ receptors. GABA_A_ receptors were expressed in *Xenopus* oocytes via injecting mRNAs prepared from rat whole brains. Results found that phytoncides potentiated a GABA_A_ receptor response suggestive of anxiolytic and sedative activity. 

### 2.31. Pimpinella peregrine (Aniseed)

The Romans, Greeks, and Egyptians cultivated *Pimpinella* spp. and used their aromatic seeds as medicine and as a condiment, and it used in Turkish folk medicine for its appetiser and tranquilising properties with most of the uses attributed to the essential oil within the seed [98]. The seeds have been traditionally used as a medicine for their analgesic, carminative, disinfectant, diuretic, and aromatic properties [166]. Inhaled *P. peregrine* essential oil was investigated for its anti-anxiety effects in scopolamine treated rats, a standard/reference drug for inducing age and dementia-related deficits. In the elevated plus maze test, scopolamine-treated rats exposed to *P. peregrine* essential oil spent more time in the open arms compared to scopolamine-treated only rats, especially at a concentration of 1%. Results imply amelioration of anxiety in scopolamine-induced rats exposed to inhalation of *P. peregrine* essential oil [98]. 

*Trans*-pinocarveol is the major compound in *P. peregrine* and has been shown to be a potent modifier of GABA_A_ receptor function as shown by its GABA-affinity of α1β2γ2 receptors expressed in transfected cell lines [124]. Thus, the anxiolytic effects of *P. peregrine* may be due to *trans*-pinocarveol working via the GABAergic system. 

### 2.32. Piper guineense

*P. guineense*, also known as African black pepper, is a perennial plant that belongs to the Piperaceae family and native to central and West Africa and is commonly used as a condiment and for cosmetic, insecticidal, and medicinal purposes possessing antibacterial, anti-inflammatory, aphrodisiac, anticonvulsant, hepatoprotective, and fertility properties [167]. Inhalation of *P*. *guineense* essential oil (derived from the fruit) was investigated in mice for its anxiolytic and sedative potential. Tankam and Ito [99] discovered that mice exposed to the inhalation of *P*. *guineense* essential oil performed positively in the behavioural tests (light-dark box and open field tests). There were increased light-dark transitions and time spent in the light compartment and a significant decrease in locomotor activity in the open field test, comparable to lavender oil. These findings demonstrate that *P*. *guineense* essential oil has anxiolytic-like and sedative effects [99]. 

Linalool and 3,5-dimethoxytoluene are main constituents in the essential oil of *P*. *guineense.* Phytochemical analysis of the essential oil derived from the dried fruit yielded 41.8% linalool and 10.9% 3,5-dimethoxytoluene [99]. Inhalation of linalool at a concentration of 4.0 × 10^−3^ mg and 4.0 × 10^−5^ mg significantly decreased locomotor activity in mice, with the latter being more potent. Inhalation of 3,5-dimethoxytoluene significantly decreased locomotor activity in mice at a concentration of 4.0 × 10^−5^ mg and 4.0 × 10^−2^ mg. These results confirm the major sedative role linalool and 3,5-dimethoxytoluene have in *P*. *guineense* essential oil [99]. Kessler et al. [124] has shown that linalool vapour significantly potentiated GABAergic currents and modulated GABA_A_ receptors in human embryonic kidney cells (HEK293 cells) and *Xenopus* oocytes. 

### 2.33. Rosa damascene (Rose)

*R. damascene*, also known as Damask rose, is a perennial bushy shrub that belongs to the Roseaceae family and is the most famous ornamental plant of the Roseaceae family worldwide, in terms of perfumery and food industries [100]. Described by Avicenna (980–1037 AD) more than a thousand years ago as medicinally beneficial for cardiac and gastrointestinal effects, cosmetic properties in eliminating the unpleasant odours, and for its antinocieptive and anti-inflammatory virtues. Pharmacologically known to possess hypnotic, analgesic, neuroprotective, anti-convulsant, cardioprotective, digestive effects, and anti-inflammatory effects [100]. 

De Almeida et al. [101] examined the anxiolytic effects of rose oil inhalation in male rats. Rats exposed to rose oil inhalation at all concentrations (1%, 2.5% and 5%) increased the number of entries in the elevated plus maze test and at concentrations of 1% and 5% increased the time spent in the open arms compared to the control group. In addition, there was an increase in the number of squares traversed in the open arms, providing an additional indication of anti-anxiety activity. The sensitivity in rats to *R. damascene* essential oil in the elevated plus maze plus diazepam challenge suggest it activates the GABA_A_ receptor complex. The use of diazepam as a positive control in the experiment supports this, as it is known to enhance GABA binding to GABA_A_-benzodiazepine receptors [51]. These results suggest that inhalation of rose oil has anxiolytic-like activity, and the authors imply the effect to be comparable to diazepam. *R. damascene* essential oil contains large amounts of the acyclic monoterpene citronellol, which has been shown to potentiate GABA_A_ receptors expressed in *Xenopus oocytes* [88] confirming GABAergic system involvement and possibly explaining a potential mode of action for its anxiolytic effects. 

### 2.34. Santalum spp. (Sandalwood) 

*S. album* is one of the eldest known perfumes with at least 4000 years of uninterrupted use and traditionally used as an incense, perfume, and embalming material in the East, possessing sedative properties [3] (p. 174). The fragrance of *S. album* L. was investigated in mice for its anti-anxiety effects. Satou et al. [102] subjected mice to a water immersion stress test followed by inhalation of *S. album* essential oil for 90 min prior to an elevated plus maze test. Results indicated anti-anxiety activity of inhalation of *S. album* essential oil under stress conditions for at least 24 h indicative of a prolonged effect. In a pilot study (*n* = 32) inhalation of Western Australian sandalwood (*Santalum spicatum* R.Br.) essential oil was evaluated for its psychophysiological effects in humans using a Triel Social Stress Test (a test where subjects deliver a speech and perform a mental arithmetic task in front of an audience [168]) and vital signs (blood pressure, heart rate, and skin conductance response). Findings revealed a reduction in blood pressure and a corresponding reduction in salivary cortisol levels compared to controls. Western Australian sandalwood essential oil therefore alleviated physiological reactions to psychological stress and facilitated recover after stress exposure [103]. It is unknown whether *Santalum* spp. or their main compounds exert anxiolytic effects via the GABAergic system. 

### 2.35. Sideritis Species

*Sideritis* species belong to the lamiaceae family and grow as herbs or small shrubs that are aromatic and have been used in folk medicine for their anti-inflammatory, antimicrobial, anticonvulsant, analgesic, and carminative properties [169]. *Sideritis* derives its name from “*sideros*” (iron), a Greek word in reference to these plants used in ancient times to heal wounds inflicted by weapons [169]. In Mediterranean countries, evening tea preparations containing *Sideritis* species have become popular due to their sedative effects. Kessler et al. [124] analysed several volatile fractions derived from *Sideritis* species to determine whether they potentiated a GABAergic response. Chemicals 1-octen-3-ol, linalool, and carvacrol significantly potentiated GABAergic currents, with the latter being the most potent modulator of GABA_A_ receptors. Kessler and colleagues also examined 13 structurally related terpenes and found in almost all cases those that contained hydroxyl groups had positive modulation on GABA_A_ receptors. This effect was further enhanced when a mono or bicyclic structure was present. Analysis of *Sideritis* species identified the presence of pinenes, and their metabolites were investigated for GABAergic activity. A significant increase in GABA-mediated currents were observed for myrtenol and verbenol [124]. In summary, terpenes derived from *Sideritis* species can modulate GABA_A_ receptors explaining their sedative effects. In addition, distinct structural patterns found within the terpenes may moderate sedative or anxiolytic mechanisms involving GABA receptors. 

### 2.36. Thymus vulgaris (Thyme)

*T. vulgaris* is one of the earliest medicinal plants used throughout the Mediterranean region familiar to Hippocrates and Dioscorides and was used by the Egyptians in the embalming process and for fumigation against infectious diseases by the ancient Greeks [3] (p. 89). It is long established as a culinary herb and widely used in Western herbal medicine to treat respiratory and digestive aliments and known for its effects on the nervous system [3] (pp. 89–90). Satou et al. [104] investigated the anxiolytic-like effects of *T. vulgaris* L. CT linalool essential oil in stress-induced mice using an elevated plus maze test. Mice that inhaled *T. vulgaris* essential oil (2 µL/L) and were subjected to a mild stressor (saline i.p. injection) significantly increased the time spent in the open arms of the elevated plus maze test. 

Mice subject to a brain fatigue model (polyinosinic-polycytidylic acid (poly I:C) injection) whilst exposed to *T. vulgaris* essential oil significantly increased the percentage of visits and the time spent in the open arms of the elevated plus maze. Thus, *T. vulgaris* essential oil induced anxiolytic-like effects in stress-induced mice [104]. The study also examined the uptake of *T. vulgaris* essential oil’s principal components linalool and terpinen-4-ol into the whole brain under experimental conditions. Findings demonstrated that stress-induced mice had significantly higher brain concentration of the monoterpene alcohols linalool and terpinen-4-ol compared to non-stress mice. Satou et al. [104] concluded that under stress conditions large quantities of *T. vulgaris* essential oil’s principle components are transferred into the brain inducing an anxiolytic-like effect. Linalool vapour has been shown to ellicit a GABAergic response in human embryonic kidney cells (HEK293 cells) and *Xenopus* oocytes via potentiating GABAergic currents and modulating GABA_A_ receptors [124]. Terpinen-4-ol has also demonstrated GABAergic system involvement by inhibiting 3-mercapto-propionic acid (3-MP), a glutamic acid decarboxylase inhibitor, induced convulsions but did not reverse flumazenil, a selective antagonist of the benzodiazepine-GABA_A_ receptor site, confirming that terpinen-4-ol does not act on the same binding site as benzodiazepines [44]. In addition, the monocyclic phenolic thymol is another chemical constituent present in large quantities in *T. vulgaris* essential oil [170] and has been shown to potentiate GABA_A_ receptors expressed in *Xenopus laevis* oocytes [163]. Collectively, these findings suggest that linalool, terpinene-4-ol, and thymol act directly or indirectly on GABAergic neurotransmission and could possibly explain a potential mechanism of action for the anxiolytic effects of *T. vulgaris*. 

### 2.37. Valeriana officinalis (Valerian)

Valerian is a flowering herb native to Asia, Europe, and North America but now grown in most parts of the world and is commonly used for medicinal purposes and has been employed as a sedative since the ancient Greeks and Romans described it. Since the late 16th century, valerian has been used to remedy insomnia and nervous conditions and was firmly established by the 18th century [105]. The effects of *V. officinalis* inhalation on sleep-wake states of rats were examined. A significant increase in pentobarbital-induced sleep duration was found following inhalation of *V. officinalis,* demonstrating sedative effects. Using rat whole brains, a GABA transaminase assay was performed to clarify the mechanism of action of *V. officinalis* inhalation. There was a significant decrease in GABA transaminase activity and an increase in GABA activity. These findings indicate that one of the ways in which *V. officinalis* exerts its action on the CNS is via the GABAergic activity changes [105]. Extracts from *V. officinalis* have shown inhibition of synaptosomal GABA uptake from mice whole brains [171] and rat brain cortices [172]. In addition, Santos et al. [172] have demonstrated valerian to induce a calcium-independent release of GABA previously accumulated in the synaptosomes. Thus, inhibition of GABA uptake and or release from nerve terminals may increase extracellular concentration of GABA in the synaptic cleft at levels high enough to activate GABA receptors [172]. Contrary to the calcium-independent GABA release findings from Santos et al. [172], Ortiz et al. [171] found valerian extract to markedly potentiate potassium stimulated release of hippocampal GABA in the presence of external calcium. Valerian extracts have also shown to potentiate benzodiazepine binding and interact with GABA_A_ receptors; however, they can also interact at other presynaptic components of GABAergic neurons [171]. Components within the essential oil of *V. officinalis* have also shown GABAergic involvement with the monoterpene borneol and is enantiomer exerting positive modulatory effects at GABA_A_ receptors [117].

### 2.38. Whisky Fragrance and Components 

Mood alterations and feelings of relaxation have been reported in humans after smelling whisky. Hossain et al. [107] examined the effects of whiskey fragrance on GABA_A_ receptors in *Xenopus* oocytes. Sedative and anxiolytic effects are seen when these GABA_A_ receptors are potentiated in the human brain. The electrical responses of GABA_A_ receptors were potentiated by whiskey fragrance, and most of the whiskey components including ethoxy, lactone derivatives, and ethyl phenylpropanoate (EPP) with the latter exhibiting the strongest effect. The researchers also examined the effects of EPP inhalation in mice injected with a GABA antagonist (pentetrazole). EPP delayed the convulsions induced by pentetrazole, implying that EPP was absorbed into the brain, where it could enhance GABA_A_ receptor responses. The same researchers investigated the effect of aged whiskey on potentiating a GABA_A_ receptor response in *Xenopus* oocytes. Findings revealed increased potentiation of a GABA_A_ receptor response with the aging period of the whiskey [108]. In addition, whiskey inhalation increased the sleeping time induced by pentobarbital in mice more than that of the same concentration of ethanol as the whiskey. Results inferring that the potentiation of the GABA_A_ receptor response and possible sedative effects are not only from the ethanol but also from the minor components in whiskey [108].

## 3. Discussion

The use of aromatic plants and oils for medicinal purposes is well established, and it is clear from the literature that aromas have known psychophysiological effects within areas of the brain associated with stress and anxiety modulation. The brain regions most crucial in regulating anxiety are a set of limbic structures, including the amygdala and hippocampus, which are intertwined and intimately connected with the olfactory neuroanatomy via extensive reciprocal axonal connections [7,8]. The amygdala and hippocampus are commonly associated in anxiety disorders, PTSD, and dementia [9,10,11]. Studies have supported the role of GABAergic neurotransmission in the amygdala in regulating anxiety-related behaviours [8]. The hippocampus has reciprocal connections with the amygdala, projects to the hypothalamus affecting the release of adrenocorticotropic hormones [10], and has been associated with significant remodeling of the GABAergic system in the dementia brain where hippocampal neurons have reduced expression of GABA_A_ receptors [12]. Neuronal inhibition of GABA is mediated via GABA receptors with GABA_A_ receptors at the forefront in controlling excitability of the brain and modulating anxiety [15]. The use of exogenous agents that bind to GABA receptors may prove a useful tool in inhibiting typical GABA-related phenotypes, such as anxiety [14]. 

Aromas found in common beverages, food, spices, volatile organic compounds, popular botanicals, and their constituents were reviewed for their anxiety reducing and sedative properties acting upon the GABAergic system. Numerous animal and human studies have confirmed anxiolytic and sedative effects from the inhalation of essential oils and or aromatic compounds [5,6,72,157]; however, only few have directly examined the effect on GABA_A_ receptors. The aromas shown to potentiate a GABA_A_ receptor response were lavender, whisky fragrance, and aged whiskey. Studies, however, have shown that oral administration of agarwood essential oil potentiating GABA_A_ receptor function and regulating GABA_A_ receptor gene expression [122] and aqueous coffee extract and coffee components elicited a GABA_A_ receptor response in *Xenopus* oocytes [137]. Despite the lack of direct studies on GABA_A_ receptors, some of the main chemical constituents present in the essential oils and/or aromatic compounds have been evaluated for their GABA_A_ receptor response. Monoterpenes (α-pinene [88,126], linalool [124], borneol [117], linalool oxide [78], thymol [163], carvacrol [124], citronellol, and hinokitiol [88]), alcohol (1-octen-3-ol [124]), lactones (jasmine lactone and lactone derivatives [78]), esters (ethyl phenylpropanoate [107] and methyl jasmonate [78]), cyclic ketone (*cis*-jasmone [78]), and ethoxy [107] have all shown to potentiate a GABA_A_ receptor response. 

Studies using pharmacological agents that interact with the GABAergic system have shown that essential oils and their constituents have GABAergic involvement. Koo et al. [46] and Komori et al. [105] evaluated the influence essential oils inhalation may have on the GABAergic system via in vivo GABA transaminase assays and GABA activity. Results confirmed that exposure to *A.* *gramineus* and *V. officinalis* inhalation inhibited GABA transaminase and raised GABA levels, confirming the role these essential oils have on the GABAergic system. In addition, Soni et al. [155] reports in their review of the medical utility of lavender that lavender exerts a similar action to benzodiazepines and increases the effects of GABA in the amygdala, although more recent studies have confirmed that the serotonergic system is involved [85]. Flumazenil, a specific GABA_A_ receptor antagonist, is used to determine anxiolytic-like occurring through the GABAergic system [43]. Co-administration of aristolen-1(10)-en-9-ol (from *N. chinensis*) and flumazenil, a specific GABA_A_ receptor antagonist, or 1,8-cineole (present in *L. Camara* and *E. globulus*) and flumazenil reversed the effects of aristolen-1(10)-en-9-ol and 1,8-cineole implying an effect on GABA_A_-benzodiazepine receptors [92,144]. Terpinen-4-ol (present in *compound anshen*, *Lavandula* spp. and *T. vulgaris*) inhibiting 3-mercapto-propionic acid (3-MP), a glutamic acid decarboxylase inhibitor, induced convulsions demonstrating GABAergic involvement but did not reverse flumazenil confirming that terpinen-4-ol does not act on the same binding site as benzodiazepines [44].

Animal studies have used various behavioural modeling to assess anxiety and the involvement of the GABAergic system. The elevated plus maze is a widely used behavioural model that has predictive validity [18,19] that is specifically suited to evaluate anxiolytic substances that act via the GABA_A_-benzodiazepine receptor complex [21,22]. More than half of the animal studies examined in this review on essential oils used the elevated plus maze test. Despite variability in the inhalation duration of the essential oil, positive results were obtained in all elevated plus maze studies except for two. The first showed no anxiolytic effect from inhalation of *I. verum*; however, its main component, *trans*-anethole, did show anxiolytic effects [81]. The second showed an anxiogenic effect from a continual two-week exposure to *C. limon* essential oil vapour [64]. It is probable that chronic exposure to the essential oil was responsible for the anxiogenic effect, as Komiya et al. [62] demonstrated anxiolytic effects in an elevated plus maze from exposure to *C. limon* essential oil vapour for 90 min. 

Of the clinical studies reviewed, there were a multitude of anxiety-related states where essential oil inhalation was applied, including pre- and post-operative anxiety, anticipation anxiety, during treatment anxiety, first-stage labour anxiety, and experimental and cognitive test anxiety. Orange [57,58], lavender [76,87], and eucalyptus [32] essential oils were used for preoperative anxiety; orange [60] and geranium [96] essential oils were used for post-operative anxiety and orange essential oil during treatment anxiety [69]; orange [59] and geranium [97] essential oils were used for first-stage labour anxiety; orange [38,69] and lemongrass [75] essential oils for experimental anxiety; and orange [68] essential oil for anticipation anxiety. There was considerable heterogeneity in the essential oil concentration, quantity applied, exposure time and mode of delivery. The concentration of the essential oil and quantity applied varied from 2–100% and 2–80 drops respectively with exposure time ranging from 3–35 min, or continual with majority of studies applied once per day, although one study was applied twice daily for three days. The mode of delivery ranged from electrical dispenser, nebuliser, to neutral support (cotton swab, medical patches, paper tissue, gauze, surgical mask, and aroma pads) with the latter making up the majority. Considering the heterogeneity of the available data from the clinical studies reviewed, further studies are warranted to confirm the application of essential oils delivered via inhalation for anxiety-related states. 

In summary, many have explored and inferred anxiety-reducing effects via bio-behavioural animal studies, a few have alluded to compounds in the essential oils that interact with GABAergic transmission, and few have conducted elegant neuropharmacology studies to show a direct binding of the compound to the GABA receptor. Further studies are warranted to confirm that aromas, essential oils, and their constituents directly interact with the GABA_A_ receptor complex.

## Figures and Tables

**Table 1:** Summary of studies on aromas associated with the GABAergic system.

Aroma	Major Components	Inhalation Duration	Subject	Test/Experiment/ Intervention	Observed Effect	Motor Activity	Mechanism of Action	Reference
*Acorus gramineus*	β-asarone euasarone α-uasarone	3 h twice daily for 7, 14 & 30 days respectively	Mice	Assays	Inhibitory CNS effects Anxiolytic-like ↑ PB induced sleep duration	Reduced	Inhibiting GABA transaminase, ↑ GABA levels, ↓ glutamate levels	[46]
*Alpinia zerumbet*	α-pinene *p*-cymene 1,8-cineole limonene	90 min	Mice	EPM LDB OFT	Anxiolytic-like	Increased		[49,50]
*Anthriscus nemorosa*	Caryophyllene trans-pinocarveol germacrene D β-elemene α-terpineol	15 min for 21 Continual days	Rats	EPM	Anxiolytic-like	Increased		[51]
*Aquilaria* spp.	Benzylacetone α-gurjunene (+)-calarene	60 min	Mice	OFT	Sedative	Reduced		[52]
*Cananga odorata*	Benzyl benzoate linalool benzyl salicylate benzyl alcohol	10 min for 7 consecutive days	Mice	EPM, LDB, OFT	Anxiolytic-like	No effect	5-HTnergic & DAnergic pathways (↑ 5-HT, ↓ DA)	[53]
*Chamaecyparis obtusa*	δ-cadinene α-pinene	90 min	Mice	EPM Stress biomarkers within the brain	Anxiolytic-like, Stress reducing	Not evaluated	↑ NGFR ↑ Arc gene expression	[54]
* Citrus aurantium *	Limonene linalyl acetate linalool	7 min	Rats	EPM, OFT, Social interaction test	Anxiolytic	No change		[55,56]
		30–35 min	Dental patients (*n* = 56)	BP, PR, RR	Anxiolytic	N/A		[57]
		30 min	CML patients (*n* = 42)	STAI BP, CF, RF	Anxiolytic	N/A		[58]
		30 min	Pregnant women (*n* = 126)	STAI Vital signs	Anxiolytic	N/A		[59]
		5 min	5 min Crack users (*n* = 51)	STAI HAS TEMP, ESC, BP, HR	Anxiolytic	N/A		[38]
		20 min 2 days after hospitilisation	ACS Patients (*n* = 140)	STAI	Anxiolytic	N/A		[60]
* Citrus bergamia *	Limonene linalool linalyl acetate	7 min	Rats	EPM, HBT	Anxiolytic Stress reducing	Increased	↓ corticosterone	[61]
*Citrus limon*	Limonene sabinene citronellal	90 min	Rats	EPM, FST, OFT	Anxiolytic-like, Sedative Stress reducing	Reduced	5-HTnergic & DAnergic pathways	[62,63]
		Continual for 2 weeks	Rats	EPM	Anxiogenic	Reduced		[64]
		25 min	Nursing students (*n* = 39)	CTAS	Anxiolytic	N/A		[65]
		Ad libitum	Nursing students (*n* = 31)	CTAS	None	N/A		[66]
* Citrus sinensis *	Limonene	5 min	Rats	EPM, LDB	Anxiolytic-like	Not evaluated		[67]
		30 min	Mice	LDB	Anxiolytic-like Sedative	Reduced		[55]
		Not specified	Dental patients (*n* = 72)	STAI	Sedative Relaxant	N/A		[68]
		30 min 2 min activation every 10 min	Child (*n* = 30) mins	Salivary cortisol PR	Anxiolytic	N/A	↓ cortisol	[69]
		5 min	Healthy males (*n* = 40)	STAI HR, EMG	Anxiolytic	N/A		[70]
*Coffee*		Not specified	Healthy women (*n* = 9)	EEG–alpha waves	Relaxant	N/A		[71]
*Compound Anshen*	D-limonene linalool linalyl acetate α-Pinene α-Santalol	60 min for 7 consecutive days	Mice	OFT Assays	Anxiolytic Sedative Hypnotic Prolonged sleep time	Reduced	↑ 5-HT ↑ GABA	[72]
*Coriander sativium*	Linalool	60 min for 21 consecutive days	Rats	EPM, FST	Anxiolytic	Increased		[20]
*Cryptomeria japonica* VOC	δ-cadinene α-murolene	30 min	Healthy male students (*n* = 16)	Salivary stress markers (α-amylase, cortisol, IgA, CgA) ECG	Relaxant Stress reducing	N/A	↓ α-amylase Inhibiting ↑ CgA	[73]
*Cryptomeria japonica*	δ-cadinene 4-epi-cubebol cubebol	30 min	Female participants (*n* = 29)	Salivary stress markers (cortisol, DHEA-s, α-amylase, CgA), POMS	Relaxant Stress reducing	N/A	↓ cortisol ↓ DHEA-s ↓ α-amylase	[74]
* Cymbopogon citratus *	Geranial neral geranyl acetate	Not specified	Healthy male graduate students (*n* = 40)	STAI SPIN Self-evaluation of tension level EMG, HR	↓ basal levels of anxiety ↓ Subjective tension	N/A		[75]
*Eucalyptus globulus*	1,8-cineole, limonene α-pinene	3 min	Cancer patients (*n* = 130)	STAI	No reduction in anxiety	N/A		[76]
		5 min	SNRB Patients (*n* = 62)	STAI POMS A-VAS BP, PR	Anxiolytic	N/A		[32]
*Forest* *Abies sachalinensis*	α-pinene camphene	90 min	Mice	EPM	Anxiolytic- Like	Not Evaluated		[77]
*Fragrant compounds from Oolong tea*	*Cis*-jasmone, jasmine lactone, linalool oxide methyl jasmonate		* Xenopus laevis * oocytes	Voltage clamp technique	↑ PB-induced sleep time	N/A	Potentiation of GABA_A_ receptor response	[78]
*Heracleum afghanicum*	Hexyl butyrate octyl acetate	60 min	Mice	OFT	Sedative	Reduced		[79]
*Hypericum scabrum*	α-pinene β-pinene myrcene	15 min for 21 continuous days	Rats	EPM, FST	Anxiolytic	Reduced		[80]
*Illicium verum*	*Trans*-anethole	90 min	Mice	EPM	No effect on anxiety	Not evaluated		[81]
*Jasminum grandiflorum*	Benzyl acetate benzyl benzoate phytol linalool	5 min	Male & female participants (*n* = 31)	EEG (α–waves) BP, PR, HR, RR, muscle potential, skin conductance, TEMP	Relaxant	N/A		[82,83]
* Lantana camara *	Sabinene 1,8-cineole	60 min	Mice	OFT	Sedative	Reduced		[84]
* Lavandula * spp.	Linalyl acetate Linalool 1,8-cineole β-ocimene terpinen-4-ol camphor	15 min	Mice	EPM, MBT	Anxiolytic-like	No effect	5-HTnergic neurotransmission–possibly via 5-HT_1A_ receptors	[85,86]
		3 min	Child dental patients (*n* = 126)	FIS BP, PR	Anxiolytic	N/A		[87]
		3 min	*Xenopus* *laevis* oocytes Cancer Pateints (*n* = 123)	Two-electrode voltage clamp technique STAI-I	No effect on anxiety	N/A N/A	Potentiated GABA_A_ receptor response	[88] [76]
* Matricaria chamomilla *	α-bisabolol oxides A & B α-bisabolol chamazulene β-farnesene	60 min twice daily for 3 days	Rats	Restriction stress Plasma ACTH	Anxiolytic effects Sedative	Not evaluated	↓ ACTH GABAergic neurotransmission	[89,90]
*Microtoena patchouli*	1-octen-3-ol, terpinolene, patchouli alcohol methyl salicylate.	20 min, 35 min	Mice	OFT	Sedative	Reduced		[91]
*Nardostachys chinensis*	Calarene aristolene	60 min	Mice	OFT	Sedative	Reduced		[92]
*Ocimum basilicum* & Living plant	Eugenol linalool methyl eugenol	60 min	Mice	OFT	Sedative	Reduced		[93]
*Ocimum basilicum*	Linalool camphor β-elemene α-bergamo-tene bornyl-acetate estragole eugenol 1,8-cineole	60 min for 21 continual days	Rats	EPM, FST	Anxiolytic	Increased		[94]
*Ocimum gratissimum*	Thymol *p*-cymene terpinene-4-ol	60 min	Mice	OFT, LDB	Anxiolytic-like Sedative	Reduced		[95]
*Ocimum sanctum*	Linalool camphor β-elemene α-bergamo-tene bornyl-acetate estragole eugenol 1,8-cineole	60 min for 21 continual days	Rats	EPM, FST	Anxiolytic	Increased		[94]
*Pelargonium graveolens*	Citronellol *trans*-geraniol	20 min for 2 consecutive Patients	Acute MI Patients (*n* = 80)	STAI	Anxiolytic	N/A		[96]
		Not Specified	Pregnant women (*n* = 100)	STAI BP, RR, PR	Anxiolytic	N/A		[97]
Phytoncides	α-pinene *cis*-3-hexenol *β*-thujaplicin		*Xenopus* *laevis* oocytes	Two-electrode voltage clamp technique		N/A	Potentiated GABA_A_ receptor response	[88]
*Pimpinella peregrine*	*Trans*-pinocarveol pregeijerene α-cubebene	15 min Rats for 21 continuous days	Rats	EPM	Anxiolytic	Increased		[98]
*Piper guineense*	Linalool 3,5-dimethoxytoluene	60 min	Mice	LDB, OFT	Anxiolytic Sedative	Decreased		[99]
* Rosa damascena *	β citronellol nonadecane, geraniol henicosane	7 min	Rats	EPM	Anxiolytic-like	Increased		[100,101]
*Santalum album*	α-santalol β-santalol	90 min	Mice	EPM	Anxiolytic	Not evaluated		[102]
*Santalum spicatum*	α-santalol β-santalol	Not specified	Female & male participants (*n* = 32)	BP Salivary Cortisol	Stress reducing	N/A	↓ Cortisol	[103]
* Thymus vulgaris *	Linalool β-myrcene terpinen-4-ol	90 min	Mice	EPM	Anxiolytic-like	No change		[104]
*Valeriana officinalis*	Isovaleric acid valerenic acid bornyl acetate	60 min	Rats	GABA transaminase assay	↑ PB-induced sleep time	Not evaluated	↓ GABA transaminase ↑ GABA	[105,106]
Whisky fragrance and components	Ethoxy lactone derivatives ethyl-phenylpropanoate		*Xenopus* *laevis* oocytes	Voltage clamp technique	↑ PB-induced sleep time	N/A	Potentiation of GABA_A_ receptor response	[107,108]

Note: 5-HT: serotonin; Arc: activity regulated cytoskeletal-associated protein; A-VAS: anxiety visual analogue scale; BP: blood pressure; CF: cardiac frequency; CGA: chromogranin A; CTAS: cognitive test anxiety scale; DA: dopamine; DHEA-s: dehydroepiandrosterone sulfate; ECG: electrocardiogram; ECS: electrical conductance of the skin; EEG: electroencephalograph; EMG: electromyography; EPM: elevated plus maze; FIS: face image scale; FST: forced swim test; HAS: humor analog scale; HR: heart rate; IgA: secretory immunoglobulin A; LDB: light dark box; MBT: marble burying test; NGFR: nerve growth factor; OFT: open field test; PB: pentobarbital; POMS: profile of mood states; PR: pulse rate; RF: respiratory frequency; RR: respiratory rate; SPIN: social phobia inventory; STAI: state-trait anxiety inventory. ↑: up regulated; ↓: down regulated.

## Data Availability

Not applicable.
